# Continuous-wave and mode-locked Bi-doped fiber laser at 1.7 μm

**DOI:** 10.1038/s41598-025-20559-9

**Published:** 2025-10-17

**Authors:** Ali Roohforouz, M. R. K. Soltanian, Pin Long, Nitika Vaish, Lawrence R. Chen

**Affiliations:** 1 Department of Electrical and Computer Engineering, McGill University, Montreal, Canada; 2 O/E Land, Inc., Lasalle, Canada; 3 Centre Énergie, Matériaux et Télécommunications Institut National de la Recherche Scientifique (INRS), Montreal, Canada

**Keywords:** Fibre lasers, Fibre optics and optical communications

## Abstract

We investigate continuous-wave (CW) and mode-locked (ML) operation of bismuth-doped fiber (BDF) lasers at 1.7 μm. We compare the slope efficiency (SE) of the Figure-of-9 (Fo9) and linear cavity configurations for CW operation and find that the linear cavity exhibits a slightly higher SE. We demonstrate that the Fo9 configuration can also support mode-locked operation. We observe multi-pulse operation and can control the number of output pulses by adjusting the pump power.

## Introduction

Bismuth doped fiber (BDF) has facilitated the development of amplifiers and lasers in the 1150–1500 nm and 1600–1800 nm wavelength ranges, owing to their host-dependent luminescence characteristics. Bi-doped germanosilicate fibers exhibit luminescence around 1450 nm^1,2^ and the luminescence window of BDFs can be extended beyond 1600 nm by using Bi-doped high-germanosilicate (up to 50 mol%) fibers^3,4^. Therefore, BDF amplifiers and BDF lasers (BDFLs) can provide amplification and lasing over a broad wavelength range to extend the operating range of optical fiber communications, sensing, and instrumentation. Specifically, BDFLs in the 1600–1800 nm wavelength range are valuable in photothermal spectroscopy for detecting gases such as methane, making them crucial for environmental monitoring and medical diagnostics^5^.

A laser operating at 1700 nm also has biomedical applications, including deep bio-imaging^6^ and skin surgery^7^. This wavelength is particularly advantageous for bio-imaging because lipids exhibit significant absorption while water shows minimal absorption, thereby enhancing imaging contrast and depth^8–10^. Finally, this wavelength is valuable in laser therapy and diagnostics, as it overlaps with the strong absorption band of water molecules, making it effective for lipolysis and other therapeutic applications^6,7^.

One of the main challenges with high-germanosilicate BDFs is their relatively low SE. The main factors that impact SE include the BDF design, cavity configuration, cavity losses, etc. Researchers have optimized the MCVD process to improve the optical properties of BDF, e.g., reducing unsaturable loss, and have demonstrated CW BDFLs around 1700 nm with high output power and slope efficiencies of 20%^11^ and 33%^12^. Other work on developing BDFLs at 1700 nm has looked at ring and dual-wavelength cavities. For example, a CW ring-cavity BDFL emitting at 1725 nm with an output power of 41 mW and a SE of 8% was demonstrated^13^ while dual-wavelength operation was achieved but with an SE of less than 1%^14^.

There is also interest in developing pulsed laser sources around 1700 nm. Bi-doped germanosilicate fibers can provide large positive group velocity dispersion (β_2_) and non-linearity, enabling mode-locking in the dispersive soliton regime. A mode-locked BDFL operating at 1700 nm was demonstrated using a figure-of-eight all-fiber design. Utilizing a 15 m long BDF, the ML laser delivered 17.7 ps pulses with net cavity dispersion of ~ 3.2 ps^2^. The pulse energy was increased to 5.7 nJ using a 100 m long BDF amplifier, although this also increased the pulse duration to 28.1 ps. Further compression of the amplified pulses to 630 fs was achieved using a 150 m long SMF-28 fiber^15^. A notable study demonstrated a linear cavity incorporating a 5 m BDF, generating 1.65 ps pulses at 1730 nm with a carbon nanotube as the saturable absorber. The broadband characteristics of the carbon nanotube enabled pulse operation in both anomalous and normal dispersion regimes of the laser cavity. In the anomalous dispersion regime, the laser delivered 1.65 ps pulses, whereas, in the normal dispersion regime, it produced 14 ps pulses, which were subsequently compressed to 1.2 ps using an external fiber compressor^16^.

Given the interest in BDFLs at 1700 nm, this paper investigates the performance of both CW and mode-locked BDFLs. In CW operation, we compare the SE of a linear cavity and Fo9 cavity while for mode-locked operation, we consider the Fo9 cavity.

## Results and discussion

We describe the performance and characteristics of the CW and mode-locked BDFLs; the details of the laser cavity configurations are provided in the Methods section.

### ASE measurements

**Fig. 1:**
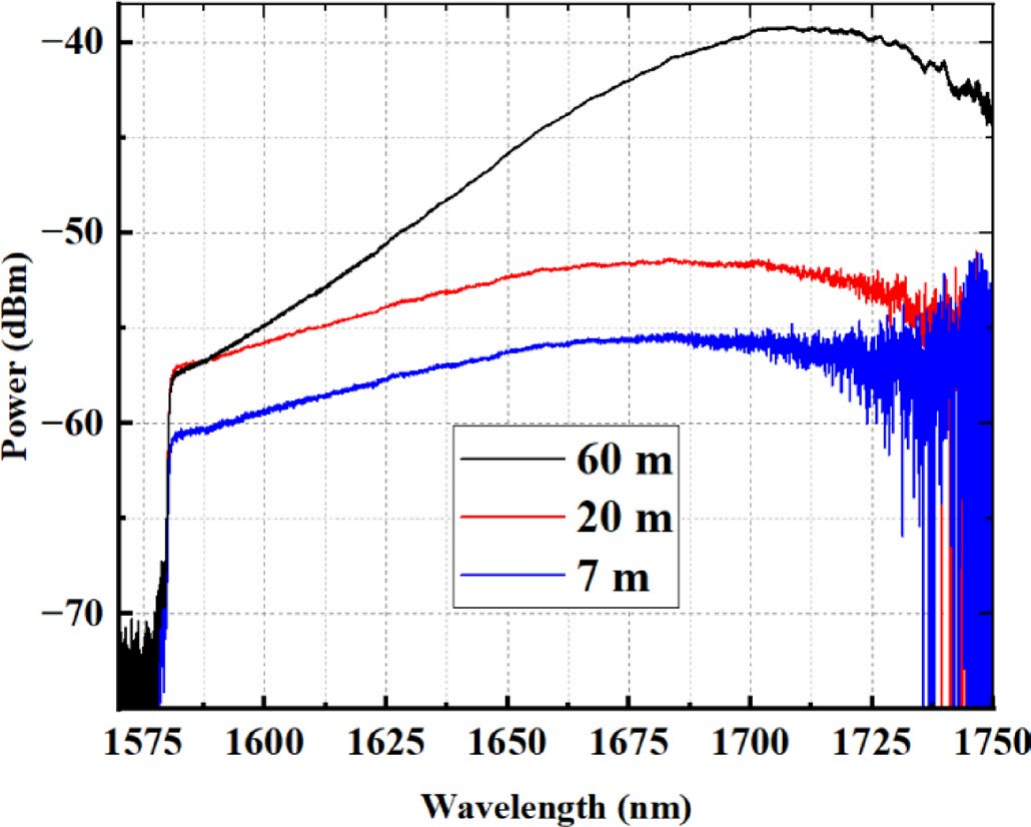
Measured luminescence spectra of the 7 m, 20 m, and 60 m high germanosilicate BDF. The spectral content beyond ~ 1725 nm appears less distinct due to our OSA exhibits low sensitivity near its upper wavelength limit (1750 nm), which reduces spectral visibility in that region.

Figure 1 shows the measured backward ASE spectra for a pump power of 600 mW launched into 7 m, 20 m, and 60 m of high germanosilicate BDF. As can be observed, the 60 m BDF exhibits a luminescence peak around 1710 nm. We can see that as the fiber length reduces, the ASE peak shifts toward shorter wavelengths, due to reabsorption of the emitted signal in a longer length of BDF.

### CW operation with the linear cavity

**Fig. 2:**
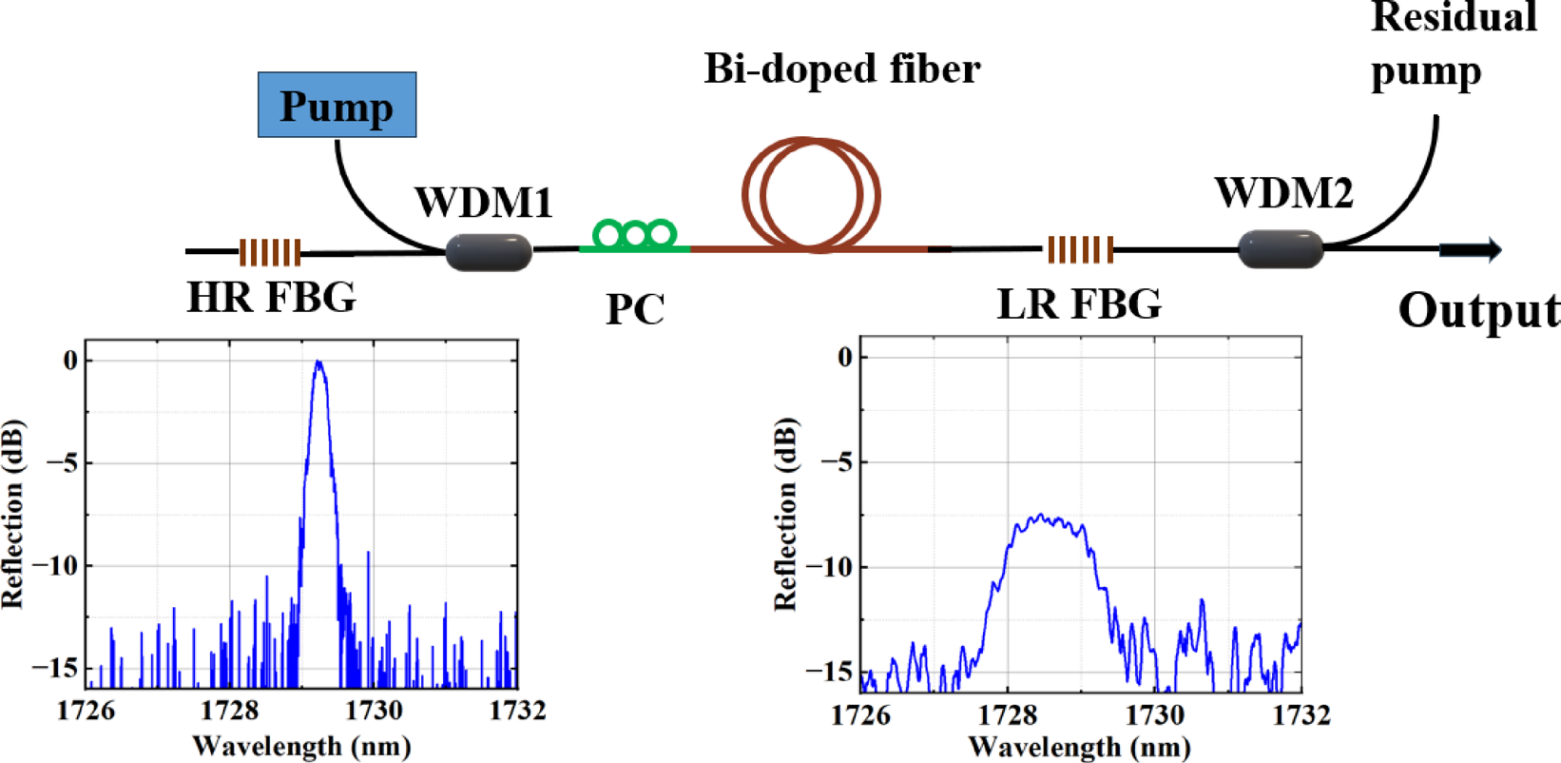
Schematic of the linear cavity configuration (the inset shows the reflection spectra of the HR and LR FBGs, with 3 dB bandwidths of 0.26 nm and 1.2 nm, and center wavelengths of 1729.3 nm and 1728.4 nm, respectively). HR FBG: high-reflectivity fiber Bragg grating (99% reflectivity), LR FBG: low-reflectivity fiber Bragg grating (18% reflectivity), PC: polarization controller, WDM: wavelength-division multiplexer.

**Fig. 3:**
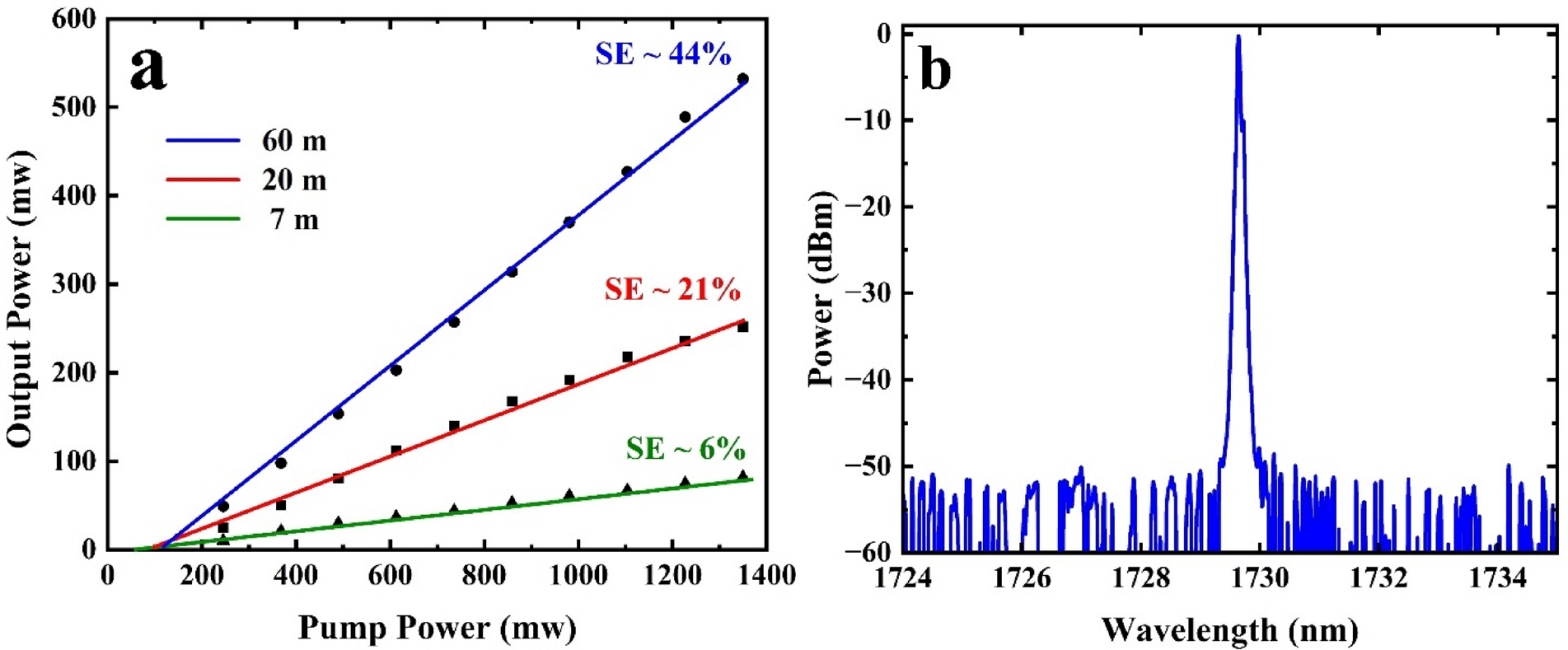
(**a**) Laser output power as a function of pump power for 60 m, 20 m, and 7 m BDF, along with the corresponding linear fit. (**b**) Laser spectrum at a pump power of 0.6 W for the linear cavity configuration with 60 m BDF.

We begin by examining the output power and SE of a linear cavity BDFL (see Fig. 2 and the Methods section for details on the laser cavity) for different lengths of gain fiber. The results are summarized in Fig. 3 for BDF lengths of 7 m, 20 m, and 60 m. It should be noted that the output power depends on the state of the polarization of light in the cavity and the polarization controller (PC) was adjusted to achieve the highest output power.

Figure 3a shows the laser output power as a function of pump power for BDF lengths of 60 m, 20 m, and 7 m, along with their corresponding linear fits. Our measured SE for 20 m BDF is consistent with the ~ 20% obtained for 15–20 m of similar BDF reported previously^11^. The intersection of each linear fit with the horizontal axis is taken as the pump threshold for the respective configuration. Accordingly, the threshold pump powers are determined to be 121 mW, 110 mW, and 52 mW for the 60 m, 20 m, and 7 m BDFs, respectively.

Figure 3b shows the typical output spectrum (using a resolution bandwidth of 0.05 nm) of the BDFL with 60 m BDF for a pump power of 600 mW. The peak wavelength is 1729.6 nm, the optical signal-to-noise ratio (SNR) is 53 dB, and the 3 dB bandwidth is 0.15 nm. The lasing spectrum is similar for BDFLs using different lengths of gain fiber. Note that the laser does not operate in a single longitudinal mode owing to the long cavity length.

**Fig. 4:**
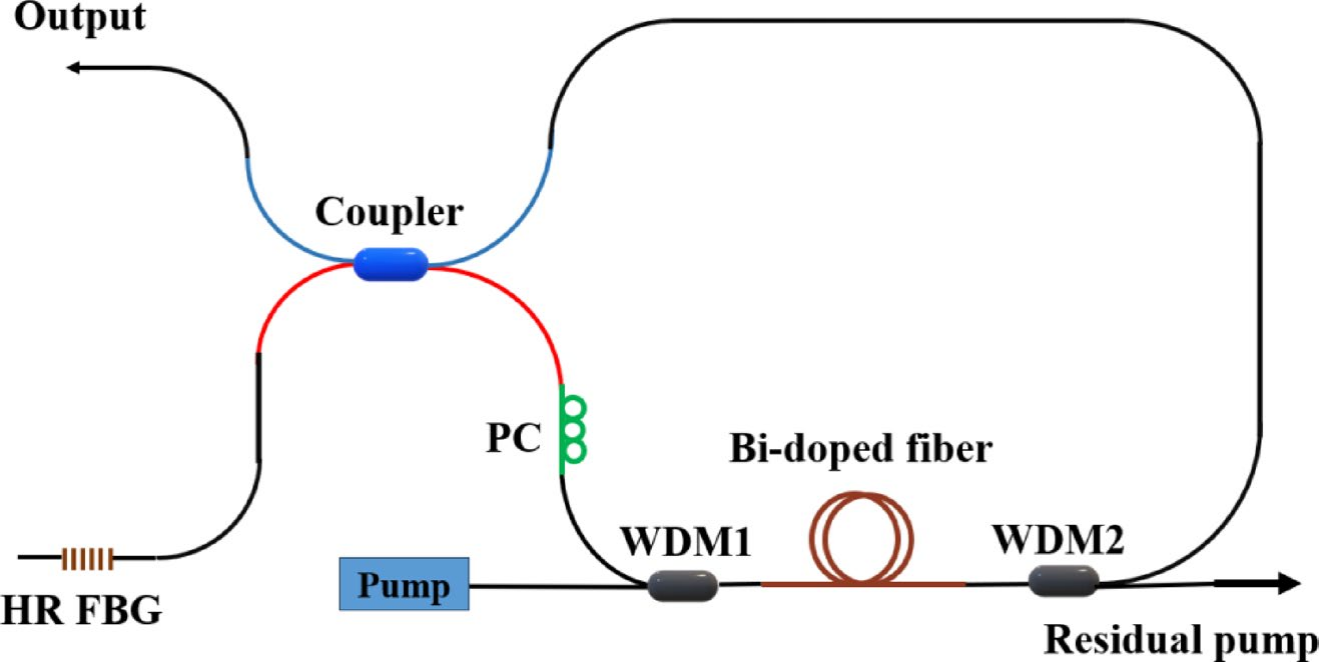
Schematic of the Fo9 laser.

### CW operation with the Fo9 cavity

**Fig. 5:**
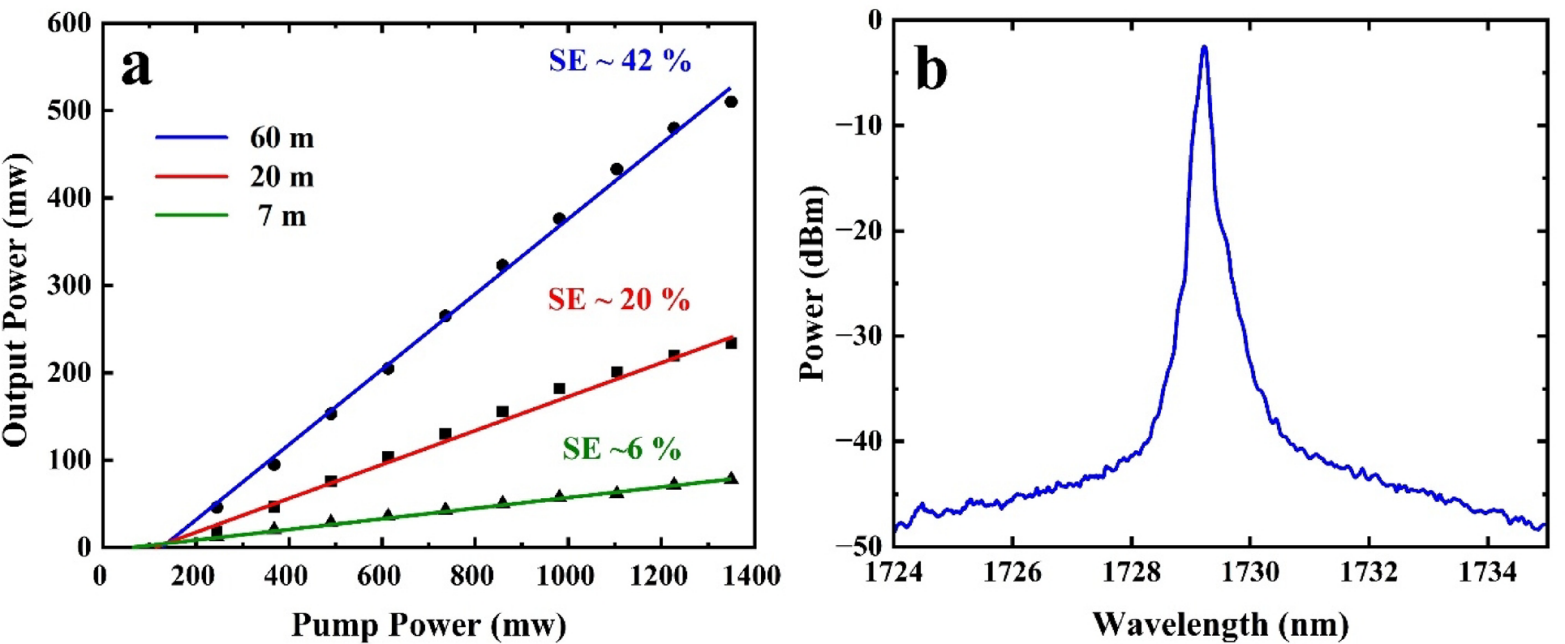
(**a**) Laser output power as a function of pump power for 60 m, 20 m, and 7 m BDF, along with their corresponding linear fit. (**b**) Laser spectrum at a pump power of 0.6 W for the Fo9 configuration with 60 m BDF.

The Fo9 cavity based on a NALM is typically used for passive mode locking. However, if the coupler ratio is highly imbalanced like in our configuration, it will not generate pulses and the laser will operate in CW mode^17^. We used a 90/10 split here that introduces a severe power imbalance. Therefore, the NALM did not maintain sufficient power in both paths to produce high modulation depth and strong saturable absorber-like behavior. Using a 90/10 reduces effective transmission modulation and therefore prevents mode locking. We also monitored the output on an oscilloscope and the temporal trace showed no periodic modulation, confirming CW operation.The output power vs. pump power and SE for the Fo9 cavity (see Fig. 4 and the Methods section for details on the laser cavity) for the same lengths of BDF are shown in Fig. 5a where again, the PC was adjusted to achieve the highest output power. The trends for output power and SE in this configuration closely resemble those observed in the linear cavity configuration. The threshold pump powers are measured using the method described in the previous section, with values of 127 mW, 113 mW, and 61 mW for the 60 m, 20 m, and 7 m BDFs, respectively. For both linear and Fo9 cavities, the threshold pump power decreases when shorter lengths of BDF are used, as shorter fibers reduce reabsorption losses. For longer lengths of BDF, there are parts that are not pumped which causes reabsorption loss. On the other hand, for both cavity configurations, longer lengths of BDF allow for higher output power. The Fo9 cavity has higher threshold pump power compared to the linear cavity for all lengths of BDF considered, indicating higher overall cavity losses. In the Fo9 cavity, both the wavelength-division multiplexer (WDM) and the output coupler contribute to higher losses compared to the linear cavity, which only has WDM loss between the FBGs and the gain fiber.

For 60-m BDF in both the linear and Fo9 cavities, the residual (unabsorbed) pump was measured ~ 3%, indicating near-complete pump absorption. Thus, ~ 60 m is near-optimal for output power and slope efficiency. Extending the fiber length beyond this adds unsaturable loss (and ASE/reabsorption penalties) with little additional pump absorption, whereas shorter fiber lengths under utilize the pump. We therefore identify ~ 60 m as an optimal active-fiber length for both configurations. For 60 m of BDF, the Fo9 has an SE of 42%, which is slightly lower (2%) than that of the linear cavity. Overall, the linear cavity has marginally better performance in terms of both SE and output power. For comparison, the SE achieved here is significantly higher than that reported for a ring cavity in^14^, where the same BDF was used. Therefore, careful cavity design such as reducing cavity loss and optimizing fiber length is critical to increasing efficiency and output power.

Figure 5b shows the typical output spectrum of the Fo9 BDFL for a pump power of 600 mW (again the laser spectrum is similar regardless of the length of gain fiber used). The center wavelength is 1729.2 nm, the optical SNR is 45 dB, and the 3 dB bandwidth is 0.16 nm, which is comparable to that of the linear cavity. One notable difference is that the Fo9 laser has a lower optical SNR (8 dB). This is primarily because the use of two wavelength selective mirrors in the linear cavity, compared to the use of a broadband mirror (loop) in the Fo9 cavity, reduced the amount of broadband emission away from the output lasing peak. Moreover, using a polarizer, the polarization extinction ratio (PER) of the laser was measured to be 17 dB suggesting that the output can be linearly polarized. To confirm the result, we also repeated the experiments for (1) different initial states of the polarization controller (PC) and (2) mode-locked operation and observed similar behavior albeit with a lower PER of 15 dB.

### Mode-locked operation with the Fo9 cavity

The Fo9 cavity generates mode-locked pulses based on the nonlinear amplifier loop mirror (NALM) concept^18^. It requires asymmetric gain for the clockwise and counterclockwise beams within the loop. These two beams, with unequal intensities, travel in opposite directions and the clockwise-propagating beam is amplified as it enters the loop while the counter-clockwise-propagating beam is amplified before exiting the loop. Both beams undergo nonlinear phase shifts due to self-phase modulation within the BDF before recombining at the coupler, leading to the formation of mode-locked pulses^18^. As we do not use polarization maintaining components the cavity may have some polarization dependence. Moreover, the gain of our BDF is strongly polarization-dependent^19^ and we observed in our CW experiments that the output power is highly dependent on the PC status. Therefore, given the polarization dependence in the cavity, the PC can be used to adjust/fine-tune the phase shifts of the clockwise and counter-clockwise beams and in particular, to obtain the conditions for mode-locking.

The 90/10 coupler is replaced with a 30/70 coupler in ML operation because when using a 90/10 coupler, one of the beams receives significantly less power, resulting in weak interference in the coupler. Although a 90/10 split increases the nonlinear phase difference, Fo9 simulations show the highest modulation depth at a splitting ratio of 0.5 and loss of ML for ratios > 0.8^17^; we therefore adopted a 30/70 coupler as a compromise that retains ample nonlinear phase difference while preserving stronger interference contrast.

**Fig. 6:**
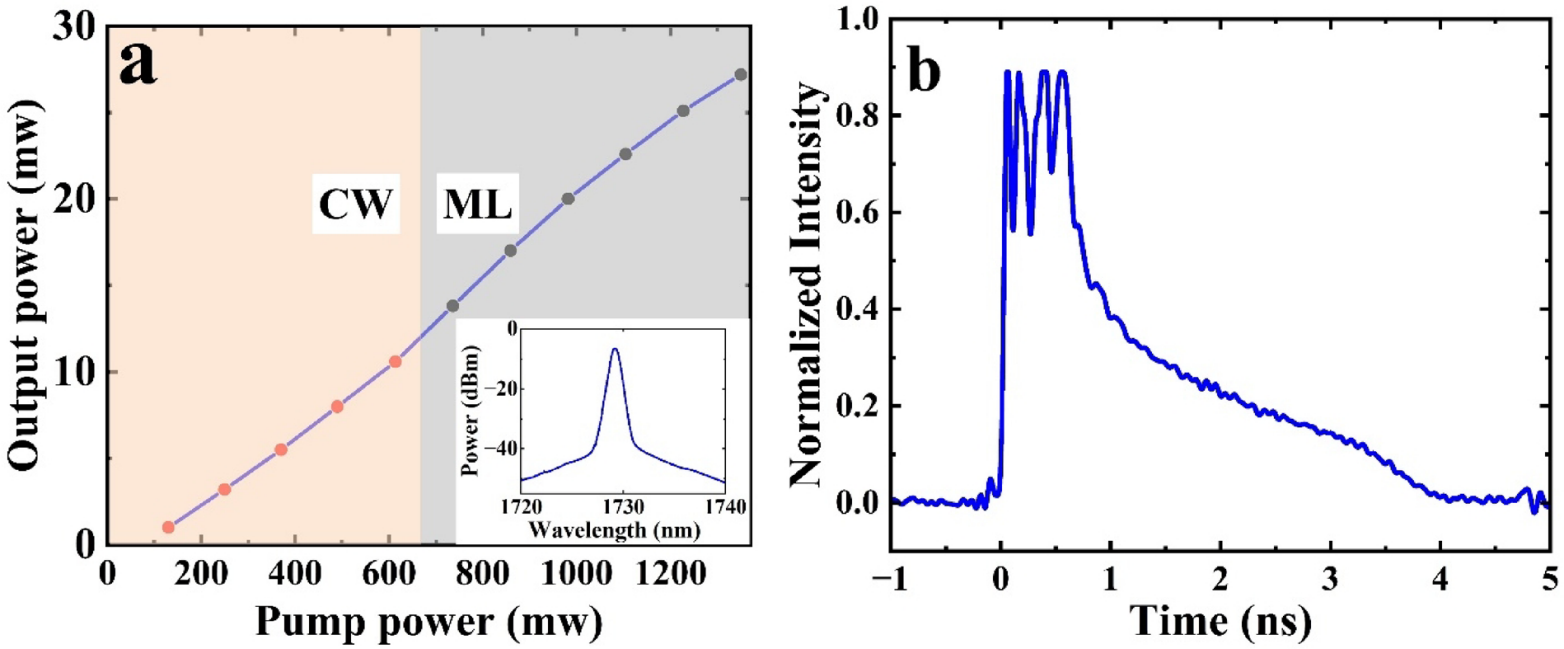
(**a**) Output power as a function of launched pump power (inset shows the output spectrum of the mode-locked operation of the laser with a 3 dB bandwidth of 0.8 nm), and (**b**) h-shaped pulse observed at a pump power of 736 mW.

As shown in Fig. 6a, mode-locking starts once the pump power is increased to 736 mW and with proper adjustment of the PC. The h-shaped temporal output shown in Fig. 6b corresponds to a multi-pulse regime, where multiple pulses circulate in the cavity simultaneously. The h-shape results from the temporal overlap of these pulses. The SE is approximately 2%, which is significantly lower than when the laser operates in CW mode. It is worth noting that the ML laser uses 27 m of BDF, compared to a similar length of 20 m in CW operation, where the SE was around 19%. The primary factor for the reduced SE is due to the change of both the coupler (from 90/10 to 30/70) and the PC state to get mode locking. As mentioned previously, the output power is highly sensitive to the state of the PC. In CW operation with Fo9 cavity, the PC was optimized to maximize output power. In contrast, for ML operation, the PC was tuned to initiate mode-locking. This PC status, while favorable for pulse formation, does not correspond to the maximum output power, which also contributes to the observed decrease in SE.

It is also worth noting that in the ML regime, we tested various fiber lengths of 20 m, 27 m, and 60 m, observing mode-locked pulses in all configurations. The most stable single-pulse operation was achieved with 27-meter fiber, making it the preferred choice for our setup.

The output spectrum of the ML laser is shown in the inset of Fig. 6a, and the 3 dB bandwidth is 0.8 nm. The measured optical bandwidth after mode-locking is significantly broader than the reflection bandwidth of the HR FBG (~ 0.26 nm). Moreover, the output bandwidth shows no significant changes (within the 0.05 nm resolution of our OSA) as the pump power was increased to the maximum available pump power of 1.35 W.

As shown in Fig. 6b, the measured temporal pulse shape resembles the so-called “h-shape”^20^, i.e., the output waveform comprises multiple pulses in the form of an h-shape where the pulse amplitudes are proportional to the number of pulses. When the pump power is increased, the number of pulses within the h-shaped waveform increases, resulting in an increase in both the amplitude and pulse width. It is important to note that this behavior is not caused by the saturation of the photodetector, as the pulse shape remains unchanged even after applying higher attenuation and reducing the input power to the photodetector.

**Fig. 7:**
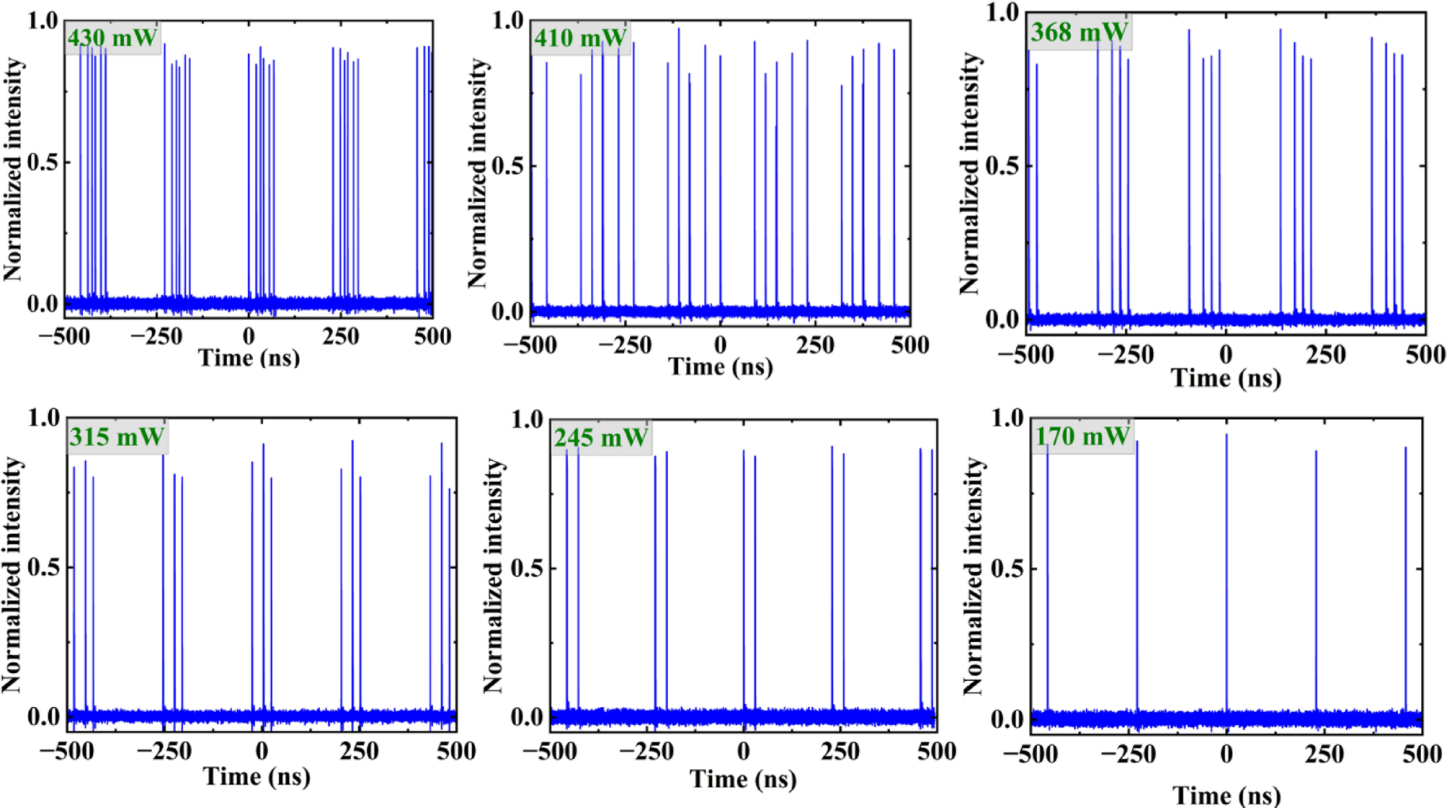
The evolution of the number of pulses as the pump power is reduced from 430 to 170 mW.

As the pump power increases beyond the ML threshold of 736 mW, we have more pulses in the h-shape; the h-shape gets longer, and the peak increases. Conversely, when the pump power is decreased below 736 mW, ML operation can still be maintained. However, the h-shape will transition to individual pulses. The number of pulses decreases with decreasing pump power all the way until 170 mW where we observe a single pulse (see Fig. 7).

**Fig. 8:**
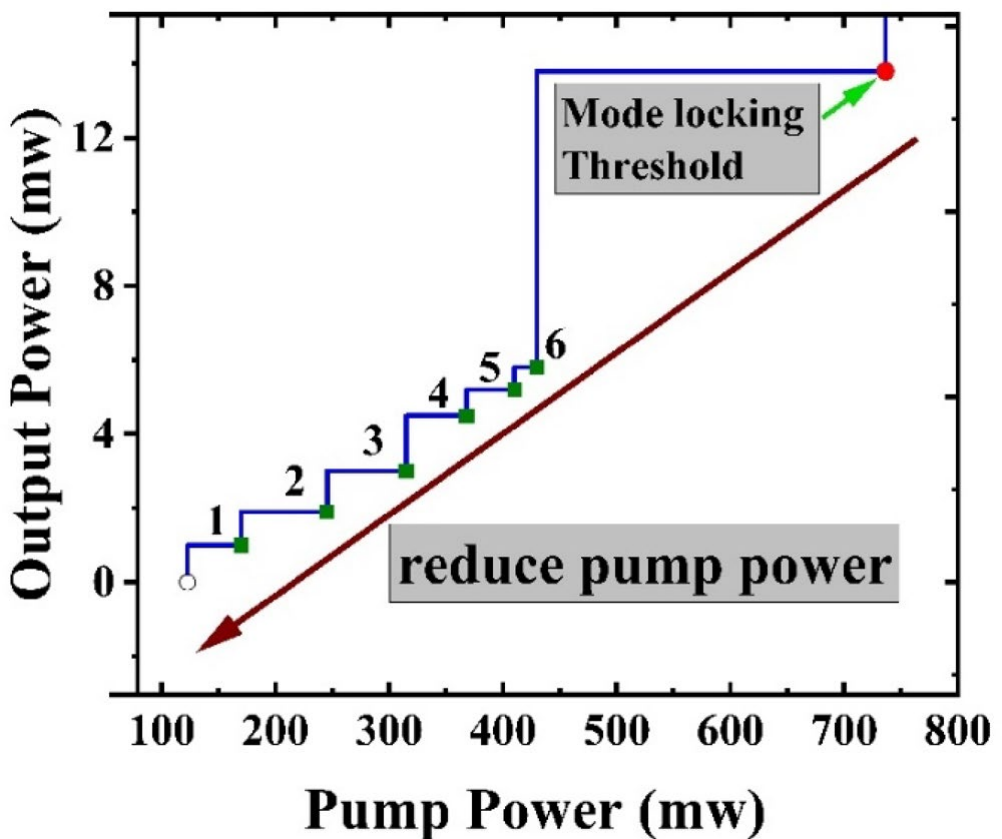
Average output power vs. pump power. The red dot marks the mode-locking threshold, and the arrow shows the pump-down sweep. The discrete numbers (1 to 6) correspond to the number of pulses observed at the corresponding pump power.

Figure 8 shows the number of pulses as a function of pump power. It shows that once we get below the threshold and transition from h-pulse to pulse ‘bursts’, there are 6 pulses which reduce to 1 once the power gets to 170 mW. As the pump power decreases below threshold, the energy in the cavity drops, and fewer pulses can be sustained, leading to the gradual disappearance of the multi-pulse structure. This is confirmed experimentally in Figs. 7 and 8, which shows a discrete reduction in pulse number, eventually transitioning to clean single-pulse mode-locking at a pump power of 170 mW.

It has been found that peak-power-clamping effects can lead to multi-pulse generation in passively mode-locked lasers^21^. This phenomenon occurs because the clamping mechanism limits the growth of peak power^22^, allowing energy to redistribute within the cavity, which can support the formation of additional pulses. Furthermore, the spectral filtering effect plays a crucial role in pulse evolution within the laser cavity, as a narrower spectrum favors multiple pulsing^23^. In our net-normal-dispersion cavity, spectral filtering trims SPM-broadened wings, shortening the chirped pulse. As a result, the available energy in the cavity can support the generation of additional pulses. In our passively mode-locked configuration, one likely cause of multi-pulsing is the use of a narrow-bandwidth FBG. Since the lasing spectrum is constrained by the HR FBG, multi-pulse formation is facilitated. The narrow bandwidth of the cavity feedback limits the maximum achievable peak power. Consequently, increasing the pump power does not increase the peak power of the pulse but instead generates additional pulses. This leads to the formation of multiple pulses within the laser cavity. Our laser’s multi-pulse behavior supports this theory, as shown in Fig. 8, where the number of pulses decreases as the pump power is reduced.

Similar dynamics, involving the generation of multi-pulses due to these mechanisms, have been experimentally demonstrated in thulium-doped fiber lasers employing a comparable Fo9 configuration and narrow-bandwidth FBGs^24^. Specifically, it has been shown that the narrow spectral filtering, combined with the peak-power clamping in our setup^22^, facilitates multi-pulse generation by redistributing cavity energy among multiple pulses at higher pump powers. Our results further support these observations, particularly the transition from h-shaped pulse structures at higher pump powers to fewer or single pulses upon reducing the pump power.

**Fig. 9:**
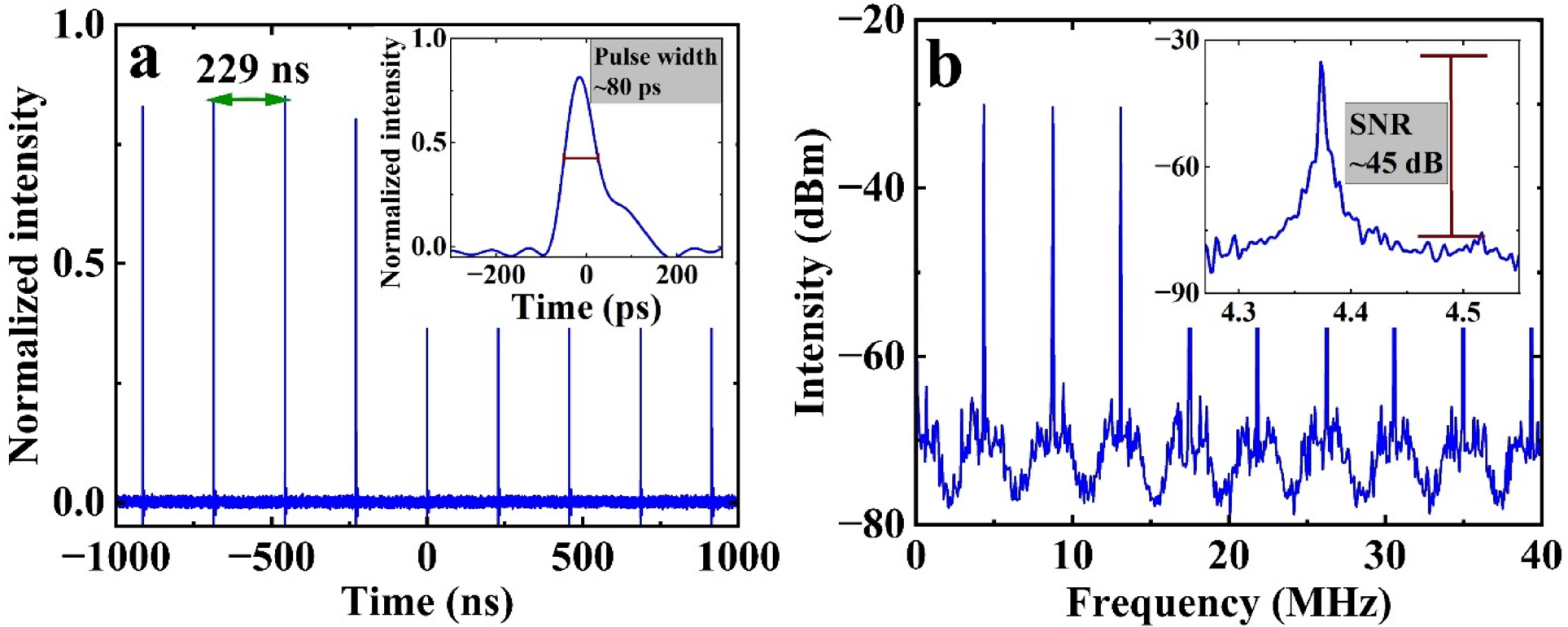
Single-pulse operation. (**a**) Oscilloscope trace of the pulse train at 170 mW pump power (inset shows a single pulse with a pulse width of 80 ps). (**b**) RF spectrum with 1 kHz resolution (inset shows the RF spectrum over a 0.3 MHz span with 300 Hz resolution).

We also characterized single pulse operation. Figure 9a shows the oscilloscope trace of the pulse train at 170 mW pump power, with an interval of 229 ns between adjacent pulses. The pulse duration, measured by a high-speed oscilloscope, is approximately 80 ps and is limited by the bandwidth of the photodetector and oscilloscope. We do not have an autocorrelator that operates at this wavelength range so cannot measure the temporal profile and duration of the output pulses more precisely. The RF spectrum of the laser, shown in Fig. 9b, indicates a repetition rate of 4.37 MHz, which corresponds to the fundamental repetition rate of the fiber laser. This frequency aligns with the cavity length of 47 m with an SNR of 45 dB.

**Summary**

We investigated the performance and characteristics of CW and mode-locked BDFLs at 1729 nm using a high-germanium-doped BDF. We demonstrate the first Bi-doped fiber laser in an Fo9 cavity at ~ 1.73 μm with both CW and mode-locked operation. The laser delivers quasi-linearly polarized output (PER ≈ 17 dB), confirming strong polarization-dependent gain of the BDF. We achieve a record 44% CW slope efficiency and showed that for CW operation, the linear cavity has slightly higher SE and provides higher output power as well as higher optical SNR. In mode-locked operation, we observe pump-tunable multi-pulsing and pump hysteresis near 1729 nm; experiments indicate that peak-power clamping together with narrow-band FBG filtering governs the multi-pulse dynamics. These empirical results may be useful for others for designing and optimizing the performance of CW and mode-locked BDFLs.

## Methods

### BDF and ASE measurement

A single-mode BDF was drawn from a preform fabricated using the modified chemical vapor deposition (MCVD) process. X-ray microanalysis of the fiber core’s chemical composition revealed a GeO₂ concentration of 35–40 mol%. The fiber developed has a cutoff wavelength of 1.2 μm, a core diameter of 2 μm, and a cladding diameter of 125 μm. The Bi-doped fiber used in this study provides optical gain from approximately 1650 nm to 1800 nm, exhibits absorption bands centered around 1400 nm and 1650 nm, and has a wavelength-insensitive unsaturable loss of ~ 0.1 dB/m across the 1500–1730 nm range. Full details regarding the BDF are available in [14]. Prior to assembling the laser, we measured the amplified spontaneous emission (ASE) spectrum. The signal was collected in a counter-propagating direction relative to the pump. The cut-off at 1575 nm in Fig. 1 is due to the WDM used to combine the pump with the output from the BDF. The spectrum was measured using an optical spectrum analyzer (OSA) with a minimum resolution of 0.05 nm and a cutoff wavelength of 1750 nm.

### Linear cavity

Figure 3 illustrates the schematic of the linear cavity configuration, which utilizes a pair of fiber FBGs at 1729 nm. The setup includes a low-reflectivity FBG (LR FBG) with a 3 dB bandwidth of 1 nm and 18% reflectivity and a high-reflectivity FBG (HR FBG) with a 3 dB bandwidth of 0.25 nm and 99% reflectivity. The pump laser is based on a 50 mW seed signal at 1552 nm which is amplified using an Er-Yb doped fiber amplifier counter-pumped with 10 W at 980 nm; it delivers a maximum output power of 1.4 W. The BDF is core-pumped by this pump source through a 1550/1700 nm wavelength-division multiplexer (WDM1), while the unabsorbed pump light is extracted through WDM2. The insertion loss of WDM and splice losses of the SMF to BDF are approximately 0.5 dB each. A PC is placed before the active fiber.

### Fo9 cavity

Figure 4 presents a schematic of a Fo9 laser. The laser configuration incorporates the previously mentioned fiber components, including HR FBG, PC, BDF, WDM, and the pump source. Moreover, A 90/10 coupler is used to direct 90% of the output, while the remaining light is used for feedback through a fiber loop that incorporates HR FBG. A PC is positioned before the active fiber again.

### Mode-locked operation mode

The 90/10 coupler is replaced with a 30/70 coupler and the length of active fiber used is 27 m. Moreover, to prevent interference with the stable mode-locked state caused by backscattered light, a polarization-independent isolator is added to the output port of the coupler. Other components including the pump source are the same as those used in the CW mode of operation. The pulse duration was measured by a high-speed oscilloscope with a 13 GHz bandwidth and an 11 GHz bandwidth photodetector (Newport 818-BB-51 F). The pigtails for all components are single-mode fiber (SMF-28e), and the total length of SMF is 20 m. The group velocity dispersion (GVD) parameter (β_2_) of SMF-28 fiber and high-germanosilicate BDF at 1700 nm are − 35 and ∼120 ps^2^/km respectively^15^. Therefore, the total cavity dispersion is estimated as a high positive of ∼ 2.54 ps^2^ indicating that the BDFL operates in a net normal dispersion region. The FBGs used are uniform (i.e., non-chirped), so the contribution of FBG dispersion to the total cavity dispersion can be neglected (we also verified negligible impact experimentally when reversing the FBG orientations with no change in results). The cavity length is about 47 m, corresponding to a fundamental repetition rate of 4.37 MHz.

## Data Availability

Data are available from the authors upon reasonable request.
